# Macrophage polarization in acute myocardial infarction: multidimensional regulation and emerging therapeutic opportunities

**DOI:** 10.3389/fimmu.2025.1710249

**Published:** 2026-01-09

**Authors:** Zilv Ye, Bo Wang, Zhengdong Wan, Jiawei Guo

**Affiliations:** 1 Department of Vascular and Endovascular Surgery, The First Affiliated Hospital of Yangtze University, Jingzhou, China; 2 Thyroid and Vascular Surgery Department, Jingzhou Hospital Affiliated to Yangtze University, Jingzhou, China; 3 Department of Pharmacology, School of Medicine, Yangtze University, Jingzhou, China

**Keywords:** acute myocardial infarction, immune modulation, inflammatory microenvironment, intervention strategies, macrophage polarization

## Abstract

Macrophage polarization is a central determinant of the inflammatory response and tissue repair following acute myocardial infarction (AMI). The dynamic transition from a pro-inflammatory M1 phenotype to an anti-inflammatory M2 phenotype is critical for limiting myocardial injury and promoting functional recovery. Recent advances have uncovered complex regulatory mechanisms governing macrophage polarization, including metabolic reprogramming, immune signaling pathways, and alterations within the cardiac microenvironment. Elucidating these processes provides important insights for the development of innovative therapeutic strategies aimed at enhancing cardiac repair. This review summarizes current knowledge on macrophage polarization in AMI, with a particular focus on the multifaceted regulatory networks that modulate the balance between M1 and M2 macrophages. Emerging therapeutic approaches targeting these pathways—such as molecular interventions, gene editing technologies, nanomedicine-based delivery systems, and modulation through traditional Chinese medicine—hold significant promise for improving cardiac repair and preventing adverse remodeling. The integration of these strategies may offer novel opportunities for the clinical management of AMI, ultimately advancing efforts to optimize heart recovery after infarction.

## 1 Introduction

Acute myocardial infarction (AMI) is a leading cause of heart failure and death worldwide, particularly among the elderly (1). According to the World Health Organization (WHO), 30% of all global deaths each year are linked to AMI, and this number is projected to surpass 23 million by 2030 (2). With the aging global population, the incidence of AMI has been increasing in recent years. Furthermore, research shows that the incidence and mortality rates of AMI are also rising among patients under 45 years of age. Early-stage atherosclerosis (AS) is the primary cause of AMI in younger individuals, with around 90% of cases resulting from plaque rupture or erosion. The remaining cases are attributed to non-atherosclerotic causes, such as spontaneous coronary artery dissection or coronary artery spasm (3). Risk factors for AMI include hypertension, hypercholesterolemia, smoking, diabetes, obesity, and a family history of cardiovascular disease. These factors, particularly in middle-aged and elderly populations, significantly elevate the risk of developing AMI (4).

AMI is typically triggered by the rupture of atherosclerotic plaques, leading to thrombosis and obstruction of blood flow in the coronary arteries, which causes myocardial ischemia and injury. As ischemia persists, myocardial cells are severely damaged, resulting in the formation of infarcted regions (5). Although the heart has limited regenerative potential, the repair mechanisms remain plastic throughout the process of cardiac repair and regeneration. This process is primarily divided into three stages: inflammation, proliferation, and remodeling. Innate immune cells, such as monocytes and macrophages, play crucial roles in tissue injury and repair following AMI (6). Macrophages, first discovered by Ilya Ilyich Mechnikov in 1882, are recognized as the first line of defense against infection and injury in vertebrates (7). After myocardial infarction, circulating monocytes are recruited to the site of injury, where they differentiate into macrophages (8). These macrophages constitute the predominant cell population during the acute inflammatory phase (9). Macrophages can secrete pro-inflammatory, anti-inflammatory, angiogenic, or reparative factors; they phagocytize dead cells; and they directly interact with other cell types to coordinate the repair response (10). The multifunctionality of macrophages is partly attributed to their diverse phenotypes and polarization states. Macrophage polarization refers to the process in which macrophages exhibit distinct gene expression profiles and functions in response to varying environmental signals. In addition to macrophages, fibroblasts, endothelial cells, and cardiac progenitor cells also contribute to the repair process (11). Fibroblasts promote fibrosis by secreting extracellular matrix, which helps reconstruct the damaged cardiac structure (12); endothelial cells restore the vascular network in the injury area through angiogenesis (13); and cardiac progenitor cells, to a limited extent, assist in myocardial cell regeneration (14). The diverse and critical roles of macrophages in each stage of repair make them the focal point of this study. Investigating their mechanisms is essential for developing novel therapeutic strategies for cardiac repair.

Macrophages demonstrate remarkable plasticity and adaptability both *in vitro* and *in vivo*. Depending on various stimuli or their environmental context, macrophages can adopt distinct phenotypes. Monocyte-derived macrophages are commonly categorized into two main phenotypes: classically activated M1 and alternatively activated M2 (15, 16). The M1/M2 paradigm was originally based on *in vitro* stimulation, surface marker expression, and the production of inflammation-related factors. LPS and IFN-γ induce M1 macrophages to secrete pro-inflammatory cytokines such as CCL3, IL-1β, IL-6, and TNF-α. IL-4 induces M2 macrophages to produce anti-inflammatory mediators, including CD206, Arg1, Fizz1, and Ym1, as well as reparative factors such as IL-10, vascular endothelial growth factor, and TGF-β1 (17). It is important to note that the M1/M2 classification oversimplifies the complex *in vivo* microenvironment. In ischemic hearts, there exists a complex combination of stimuli that drive both M1 and M2 activation. Macrophage subsets can be further subdivided based on the specific stimuli they encounter *in vitro*. For example, stimulation via Toll-like receptors classifies M1 macrophages into the M1a subset, whereas stimulation with high-mobility group box 1 (HMGB1) identifies the M1b subset. Similarly, M2 macrophages can further differentiate into M2a type when exposed to IL-4 or IL-13, M2b type in response to immune complexes and IL-1β, and M2c type when stimulated by IL-10, TGF-β, or glucocorticoids (18). These subsets also exhibit distinct physiological properties; for example, M1b macrophages demonstrate weaker phagocytic activity than M1a macrophages. M2a and M2c macrophages primarily regulate adaptive immune responses, while M2b macrophages are involved in the suppression of inflammation (19). The M4 phenotype, on the other hand, refers to monocytes exposed to CXCL4 (20). Additionally, these macrophage phenotypes can interconvert under specific *in vitro* conditions, and changes in their polarization states at various stages directly impact heart recovery and functional repair (21).

Although the M1/M2 framework offers a useful foundation for understanding macrophage functions, it oversimplifies the complexities of macrophage polarization, particularly in complex immune environments like AMI. Macrophages do not merely exist as M1 or M2 phenotypes; instead, they may exhibit a range of mixed or intermediate phenotypes depending on the immune microenvironment. For instance, phenotypes such as M2a and M2b, discussed earlier, demonstrate both pro-inflammatory traits and reparative functions. These complex polarization states cannot be fully captured by the traditional M1/M2 framework, which fails to account for the influence of factors such as metabolic reprogramming, epigenetic modifications, and other environmental influences on macrophage functional states. Indeed, macrophage polarization is a dynamic, multidimensional process governed by numerous signaling pathways, particularly those involved in metabolic shifts, such as glycolysis and fatty acid oxidation, which play crucial roles in the transitions between polarized states. As a result, the M1/M2 framework does not fully encompass the diversity and transitional aspects of this process, which limits our understanding of the macrophage’s complex role in cardiac repair.

However, for the purposes of this article, we maintain the use of the M1/M2 framework. This is because it offers a clear and simple structure that allows us to relate macrophage polarization in AMI to immune functions in cardiac repair. While this framework does not fully capture the complexity of macrophage function, it provides an effective way to illustrate the functional transitions between the inflammatory phase (predominantly M1) and the reparative phase (predominantly M2), especially in the context of macrophage role transformation following AMI. Furthermore, the M1/M2 framework serves as a foundational tool for future research, enabling a focus on the most basic functional categories within the larger, more complex network of signaling mechanisms. Although it simplifies the polarization process, the M1/M2 framework remains a useful starting point for studying macrophage functions in cardiac repair, particularly in the context of large-scale immune responses, offering a practical theoretical basis for clinical interventions. Therefore, while we acknowledge the framework’s limitations, its utility in structuring the discussion of macrophage polarization and helping readers understand the functional roles of different polarization states justifies its continued use in this article. Future research should move beyond this framework to explore more complex, multidimensional models of macrophage polarization—particularly those that consider intermediate phenotypes, metabolic reprogramming, and the interactions between these processes and immune responses—thereby providing a more comprehensive understanding of macrophage functions in cardiac repair and immune responses.

Macrophages in infarcted hearts display significant heterogeneity. In recent years, an increasing body of research has highlighted the dual role of macrophages in cardiac repair (22–24). During the first three days after infarction, M1 macrophages predominate. These inflammatory M1 macrophages secrete cytokines, chemokines, growth factors, and matrix metalloproteinases (MMPs) to assist in the removal of cellular debris and the degradation of the extracellular matrix (25). However, the persistent presence of M1 macrophages contributes to the expansion of the infarct area and impedes the resolution of inflammation and scar formation. As the repair process advances, by days 5 to 7, anti-inflammatory M2 macrophages take over and promote tissue repair. M2 macrophages produce factors such as IL-10, vascular endothelial growth factor, and TGF-β1, which are involved in anti-inflammatory responses, angiogenesis, and tissue repair. Additionally, M2 macrophages phagocytose apoptotic cells, aiding in neovascularization and scar tissue repair (18, 26, 27). The shift from M1 to M2 macrophages contributes significantly to the improvement of repair and functional recovery after AMI. It is important to note that macrophage polarization is influenced not only by the type of polarization but also by the immune environment, metabolic state, and dynamic changes within the cardiac microenvironment. This process exhibits both temporal and spatial specificity (24, 28), a topic that will be further explored in the following sections.

Despite significant advances in understanding the mechanisms underlying macrophage polarization, including metabolic reprogramming, epigenetic regulation, and interactions with the cardiac microenvironment and other immune cells, the specific role of macrophages in tissue repair following AMI remains poorly defined. Current research primarily focuses on the roles of M1 and M2 macrophage polarization; however, systematic analyses of polarization transitions, temporal regulation, and dynamic changes across diverse immune environments are still limited. Additionally, while therapeutic strategies such as immune modulators, gene therapy, and biomaterial-based targeted delivery systems have shown promise in preclinical studies, these approaches are still in the exploratory phase and require further validation regarding their efficacy and clinical applicability.

This review systematically summarizes the role of macrophage polarization in tissue repair following AMI, with a focus on its underlying regulatory mechanisms. Specifically, it examines the multidimensional regulation of macrophage polarization, including the interplay between metabolic reprogramming, immune factors, and the cardiac microenvironment. Furthermore, we provide an overview of current therapeutic strategies targeting macrophage polarization, such as immune modulators, gene editing, nanomedicine-based delivery systems, and traditional Chinese medicine interventions. By summarizing recent research and emerging technologies, this review offers novel insights and therapeutic strategies for immune modulation in cardiac repair following AMI.

## 2 Metabolic and epigenetic regulation of the time window in macrophage polarization

### 2.1 The inflammatory microenvironment induced by AMI and the time window for macrophage polarization

AMI induces sterile inflammation, characterized by the recruitment and activation of both innate and adaptive immune system cells (29). The immune microenvironment undergoes significant changes after AMI, and it plays a crucial role in guiding the polarization of monocytes and macrophages (30). The infarct microenvironment is initially dominated by pro-M1 mediators, such as IFN-γ and GM-CSF, followed by pro-M2 factors like IL-10 and TGF-β1, which may influence macrophage polarization (31, 32). Specifically, macrophage polarization is primarily regulated by damage-associated molecular patterns (DAMPs), hypoxia-inducible factors, and the dynamic modulation of pro-inflammatory and anti-inflammatory cytokines (33). Cell death resulting from myocardial infarction releases a large amount of DAMPs, such as high-mobility group box 1 (HMGB1), extracellular ATP, and uric acid crystals, which activate pattern recognition receptors (PRRs) on macrophage surfaces, such as Toll-like receptor 4 (TLR4) and receptor for advanced glycation end products (RAGE), initiating macrophage inflammation (34). At this stage, macrophages predominantly polarize to the M1 type, secreting pro-inflammatory factors (35), intensifying local inflammation and contributing to expanded myocardial damage (18).

Hypoxia is another critical factor regulating macrophage polarization after AMI. Following myocardial infarction, local hypoxia in the injured tissue stabilizes hypoxia-inducible factor-1α (HIF-1α), which activates the expression of genes related to pro-inflammatory cytokines. The activation of HIF-1α not only enhances M1 macrophage polarization directly but also exacerbates the pro-inflammatory response by promoting glycolysis and lactate accumulation, further amplifying M1 polarization during the acute phase (36). Over time, metabolic reprogramming and the gradual upregulation of key anti-inflammatory factors facilitate the transition of macrophages towards the M2 phenotype. In this persistently hypoxic microenvironment, the expression and activity of PKM2 are regulated downstream of HIF-1α (37). PKM2 plays a central role in coordinating metabolic reprogramming by balancing glycolysis and oxidative phosphorylation, thus promoting the shift from the pro-inflammatory M1 phenotype to the reparative M2 phenotype. The metabolic alterations induced by PKM2, together with cytokine regulation, lead to the secretion of anti-inflammatory mediators by M2 macrophages. These mediators contribute to tissue repair, suppress inflammation, and promote tissue remodeling during the later stages of AMI recovery (38, 39).

The regulation of the time window is critical in macrophage polarization. The innate immune response plays a key role in the AMI response, encompassing three stages: inflammation, proliferation, and maturation. Monocytes and macrophages are central to all three stages (25). During the inflammatory phase following AMI, endothelial cells increase the expression of intercellular adhesion molecule 1 (ICAM-1) and vascular cell adhesion molecule 1 (VCAM-1), while histamine released by mast cells enhances vascular permeability. These changes together promote the infiltration of leukocytes. Various effector cells, including neutrophils and monocytes, are subsequently attracted to the injured area (29). Neutrophils are the first to migrate to the infarct site, while circulating monocytes begin to infiltrate the infarcted area approximately 30 minutes after myocardial infarction. In the early stages, the number of monocytes surpasses that of neutrophils. The majority of macrophages recruited to the ischemic region originate from monocytes in the peripheral blood, which mainly come from the bone marrow and spleen (19). The recruitment of monocytes is dependent on the activation of the CCL2/CCR2 signaling pathway. Once in the damaged tissue, monocytes differentiate into M1 macrophages, which perform essential functions such as clearing necrotic cells, secreting pro-inflammatory factors, and initiating local immune responses (25, 40). Notably, during the first three days after infarction, most of the macrophages in the infarcted area are derived from the recruitment of peripheral blood monocytes, with minimal renewal of resident cardiac macrophages.

The inflammatory phase concludes when M1 macrophages phagocytose neutrophils, marking the transition into the proliferative phase (41). During this phase, M1 macrophages reduce the production of pro-inflammatory factors and begin to increase the secretion of two key anti-inflammatory factors: IL-10 and transforming growth factor β (TGF-β). This shift signals the onset of M2 polarization. M2 macrophages, which play a role during this stage, promote angiogenesis by secreting IL-10, TGF-β, and vascular endothelial growth factor (VEGF), and also activate fibroblasts (18). TGF-β stimulates newly differentiated myofibroblasts to secrete collagen (primarily type III), fibronectin, and other extracellular matrix components. Moreover, macrophages regulate matrix turnover by modulating matrix metalloproteinases (MMPs) and tissue inhibitors of metalloproteinases (TIMPs) (23). These processes result in the formation of a temporary collagen matrix, rich in fibrin and fibronectin. The activation of M2 macrophages is critical for the smooth transition from the acute inflammatory phase to the repair phase. An imbalance between M1 and M2 macrophages may lead to unsuccessful cardiac remodeling, contributing to myocardial fibrosis (36).

Maturation is the terminal phase of post–myocardial infarction cardiac repair. It is characterized by collagen cross-linking, cessation of angiogenesis, and clearance of fibroblasts and leukocytes from the previously injured territory. ECM remodeling underpins this phase and ultimately yields a dense, acellular fibrotic scar (5). The maturation phase can persist for months after the proliferative phase and may be accompanied by low-grade inflammation, which drives cardiomyocyte apoptosis, depresses contractile function, and promotes further ECM remodeling.

Therefore, the polarization process of macrophages in AMI is highly dynamic, with the time window of polarization significantly impacting the final outcome of cardiac repair. Understanding the time window and its regulatory mechanisms offers potential targets for future therapeutic interventions to promote cardiac repair and prevent adverse remodeling.

### 2.2 Metabolic reprogramming and polarization bias

Macrophages exhibit distinct metabolic characteristics in different functional states, a process known as metabolic reprogramming. During the repair process after AMI, metabolic reprogramming plays a critical role in shaping macrophage function and driving the shift from inflammation to repair. Studies confirm that altering macrophage metabolism drives the transition between pro-inflammatory (M1) and repair (M2) phenotypes (42).

**Figure 1:**
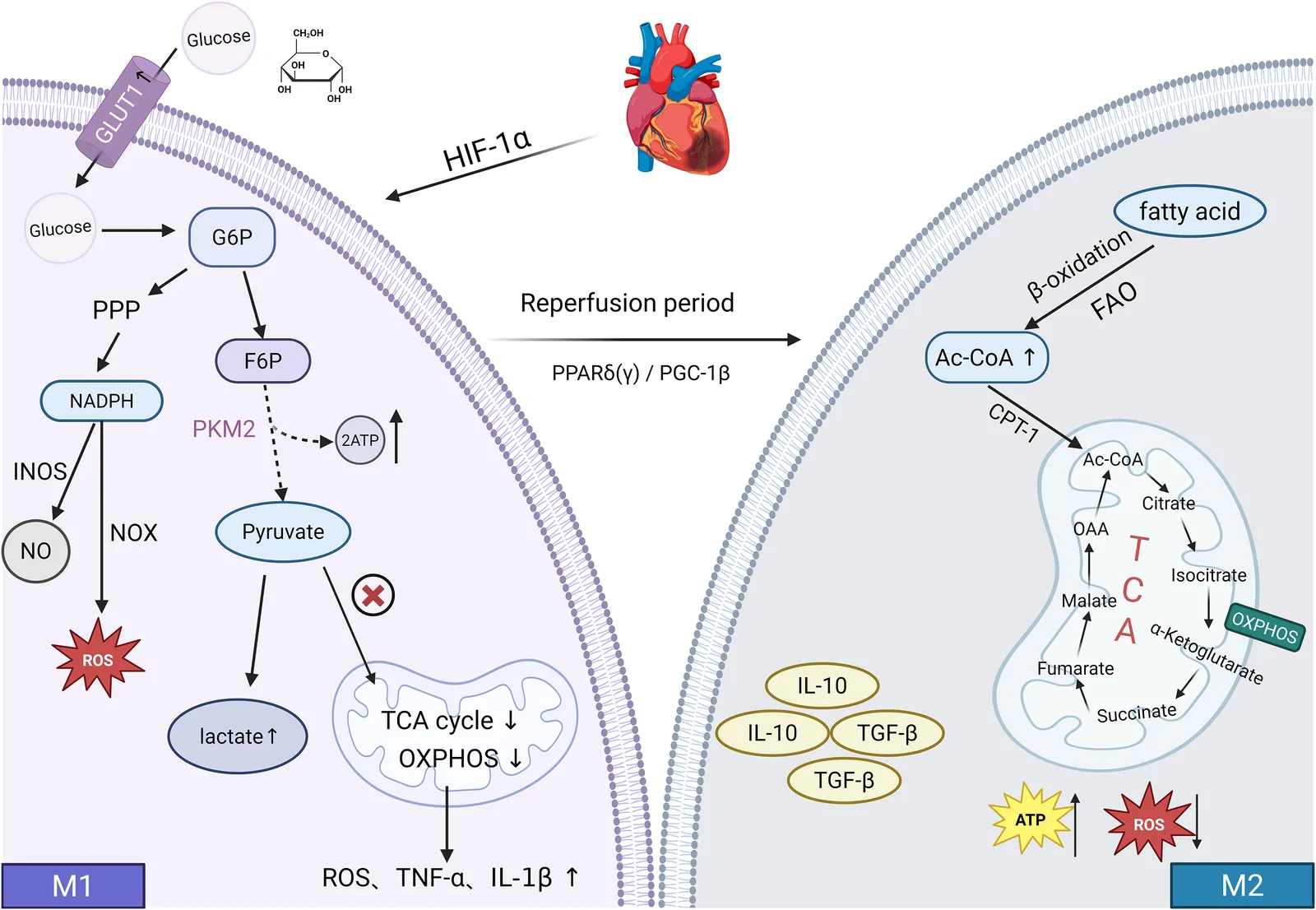
The metabolic reprogramming and polarization of macrophages during the repair process following AMI. During the ischemic phase (M1), macrophages switch to glycolysis, a process driven by HIF-1α, which leads to increased ROS production and heightened inflammatory responses. Both the TCA cycle and OXPHOS are suppressed, while lactate production rises, promoting the secretion of pro-inflammatory cytokines. Upon entering the reperfusion phase (M2), M2 macrophages maintain their anti-inflammatory repair functions through FAO and the normal tricarboxylic acid cycle. These processes rely on OXPHOS for ATP generation, reducing ROS accumulation. The transition from M1 to M2 is influenced by the oxygen recovery during reperfusion and is further regulated by lactate, ROS, and local metabolic factors.

M1 macrophages predominantly rely on glycolysis for energy metabolism. The hypoxic environment of myocardial infarction, regulated by HIF-1α, induces a shift in macrophages from oxidative phosphorylation (OXPHOS) to glycolysis (43)This metabolic mode rapidly generates ATP and lactate, sustaining high pro-inflammatory activity. Simultaneously, the TCA cycle is inhibited, and OXPHOS activity decreases (44), further enhancing the production of inflammatory factors, and reactive oxygen species (ROS) (43, 45)(**Figure 1**). Glycolytic intermediates enter the pentose phosphate pathway (PPP), generating NADPH for iNOS-catalyzed nitric oxide production and NADPH oxidase-mediated ROS generation, thus enhancing cytotoxic effects (44, 46). However, persistent M1 polarization may prolong myocardial injury and delay repair.

In contrast, M2 macrophages rely on fatty acid oxidation (FAO) and a complete TCA cycle to maintain anti-inflammatory and repair functions (47, 48). During the repair phase, M2 macrophages convert long-chain fatty acids into acetyl-CoA via FAO, which enters the TCA cycle to generate ATP through OXPHOS, reducing ROS accumulation and maintaining a low cytotoxic environment. This environment promotes angiogenesis, matrix remodeling, and fibrosis inhibition (49)(**Figure 1**). Citrate generation not only supports energy metabolism but also provides substrates for the biosynthetic processes required for tissue regeneration (50). However, under the hypoxic conditions of AMI, sustained FAO and OXPHOS may be limited, and the mechanisms by which M2 macrophages maintain repair functions in low oxygen remain unclear.

The temporal dynamics of AMI further complicate macrophage polarization. During the ischemic phase, hypoxia significantly induces HIF-1α upregulation, driving the dominance of M1 glycolysis (51). The changes in oxygen tension during reperfusion partially restore oxidative metabolism, creating a transition window for metabolic reprogramming that favors the M1 to M2 shift (43). However, this transition is influenced by local metabolic factors such as lactate, ROS, and nutrient supply, which may either promote or inhibit it (52, 53). This suggests that the metabolic polarization dynamics in AMI are not static or binary but are influenced by fluctuating oxygen gradients and metabolic signals.

It is important to note that most existing models classify macrophages as either M1 or M2, but *in vivo* evidence indicates the presence of mixed or intermediate phenotypes with coexisting metabolic programs at the infarct border zone. This heterogeneity presents both a challenge and an opportunity, suggesting that future interventions should focus on specific metabolic checkpoints rather than simply promoting the dominance of one phenotype.

### 2.3 Epigenetic regulatory mechanisms

The term “epigenetics” was first introduced by C.H. Waddington in 1942 (54), and in 2008, the scientific community reached a consensus definition: “Epigenetic traits are stable genetic phenotypes arising from chromosomal changes without altering the DNA sequence” (55). Epigenetic mechanisms, including DNA methylation, histone modifications, non-coding RNAs (ncRNA), and chromatin remodeling, play a central role in macrophage polarization. These mechanisms regulate gene expression without changing the DNA sequence (56). In AMI, these modifications regulate macrophage function, balancing the inflammatory response and tissue repair.

Histone modifications are among the most important forms of epigenetic regulation, encompassing various types such as methylation, acetylation, ubiquitination, phosphorylation, and SUMOylation. These modifications directly influence chromatin structure and gene accessibility. The most widely studied and significant types of histone modifications are histone methylation and acetylation (57). M1 macrophages typically obtain energy through glycolysis, and their histone modifications are marked by the activation of pro-inflammatory gene promoters. These modifications promote the expression of pro-inflammatory genes, enhancing the pro-inflammatory functions of M1 macrophages (58). In contrast, M2 macrophages rely on fatty acid oxidation and the TCA cycle for energy metabolism. The related histone modifications usually signify the silencing of anti-inflammatory and reparative genes, assisting macrophages in executing repair and anti-fibrotic functions (59). Notably, histone lactylation, an emerging epigenetic modification, has attracted significant attention in recent years for its role in macrophage polarization (60). In the hypoxic environment of AMI, lactylation promotes the polarization of M2 macrophages, supporting their anti-inflammatory and reparative functions (61).

Non-coding RNAs (ncRNAs), including microRNAs (miRNAs) and long non-coding RNAs (lncRNAs), do not directly alter chromatin structure but play essential roles in post-transcriptional regulation of gene expression. They are significant regulators of macrophage polarization (56). By binding to mRNA or interacting with chromatin, these ncRNAs modulate gene expression profiles, influencing macrophage function and playing crucial roles in cardiac repair after AMI. miRNAs are small, single-stranded RNA molecules that typically regulate gene expression by binding to the 3’ untranslated region (UTR) of target mRNAs, leading to mRNA degradation or translation inhibition (62). Studies have shown that miRNAs play key roles in macrophage polarization, particularly in immune responses following AMI (63). lncRNAs, RNA molecules longer than 200 nucleotides that generally do not encode proteins, regulate macrophage polarization through several mechanisms (64), including chromatin remodeling, interaction with miRNAs, and direct binding to transcription factors (65).

DNA methylation and chromatin remodeling are fundamental mechanisms of epigenetic regulation. Historically, DNA methylation was considered stable, but with the discovery of the TET family of enzymes, DNA methylation has been shown to be reversible. It can regulate gene expression by converting 5-methylcytosine (5mC) to 5-hydroxymethylcytosine (5hmC) and its further metabolites (66). M1 macrophages promote inflammation in a hypomethylated state of pro-inflammatory genes, whereas M2 macrophages inhibit excessive inflammation through hypermethylation of repair genes (67–69). Thus, regulating DNA methylation through enzymes offers potential new therapeutic strategies for AMI (56, 70). Chromatin remodeling, on the other hand, regulates gene accessibility by altering chromatin conformation. Chromatin remodeling factors, such as the SWI/SNF complex and BRG1, play vital roles in macrophage polarization transitions (71, 72). These factors influence the openness and tightness of chromatin, promoting transcription of pro-inflammatory genes in M1 macrophages, thus triggering immune responses. In the M2 polarization and repair phase, chromatin remodeling regulates the expression of genes associated with tissue repair and anti-fibrosis, supporting the repair process (73, 74). While DNA methylation and chromatin remodeling play important roles in macrophage polarization, their specific mechanisms in AMI remain to be explored in greater detail compared to histone modifications and ncRNAs.

These epigenetic regulatory mechanisms play critical roles in macrophage polarization and offer potential directions for AMI treatment strategies. Given the constraints of space and content, this section has only outlined the basic principles and roles of these mechanisms, aiming to provide readers with a diverse array of therapeutic approaches. The subsequent section on signaling pathways will explore in more depth the specific roles of macrophage regulatory mechanisms and their regulatory networks in AMI.

## 3 Signal pathway-mediated macrophage polarization mechanisms

In AMI, macrophage polarization is regulated not only by the inflammatory microenvironment, metabolic reprogramming, and epigenetic factors but also by a series of signaling pathways. The inflammatory microenvironment provides the initial signals for macrophage polarization, while metabolic reprogramming and epigenetic regulation further drive the formation of polarization bias. At the same time, signaling pathways play a crucial role in polarization by regulating macrophage function and status, determining the strength of the immune response and repair capacity. Understanding the mechanisms of these signaling pathways not only deepens our understanding of the multifaceted regulatory roles of macrophages in AMI but also provides a theoretical foundation for exploring new therapeutic strategies. Next, we explore how several key signaling pathways regulate macrophage polarization after AMI, balancing cardiac repair and the immune response.

### 3.1 NF-κB signaling pathway

**Figure 2:**
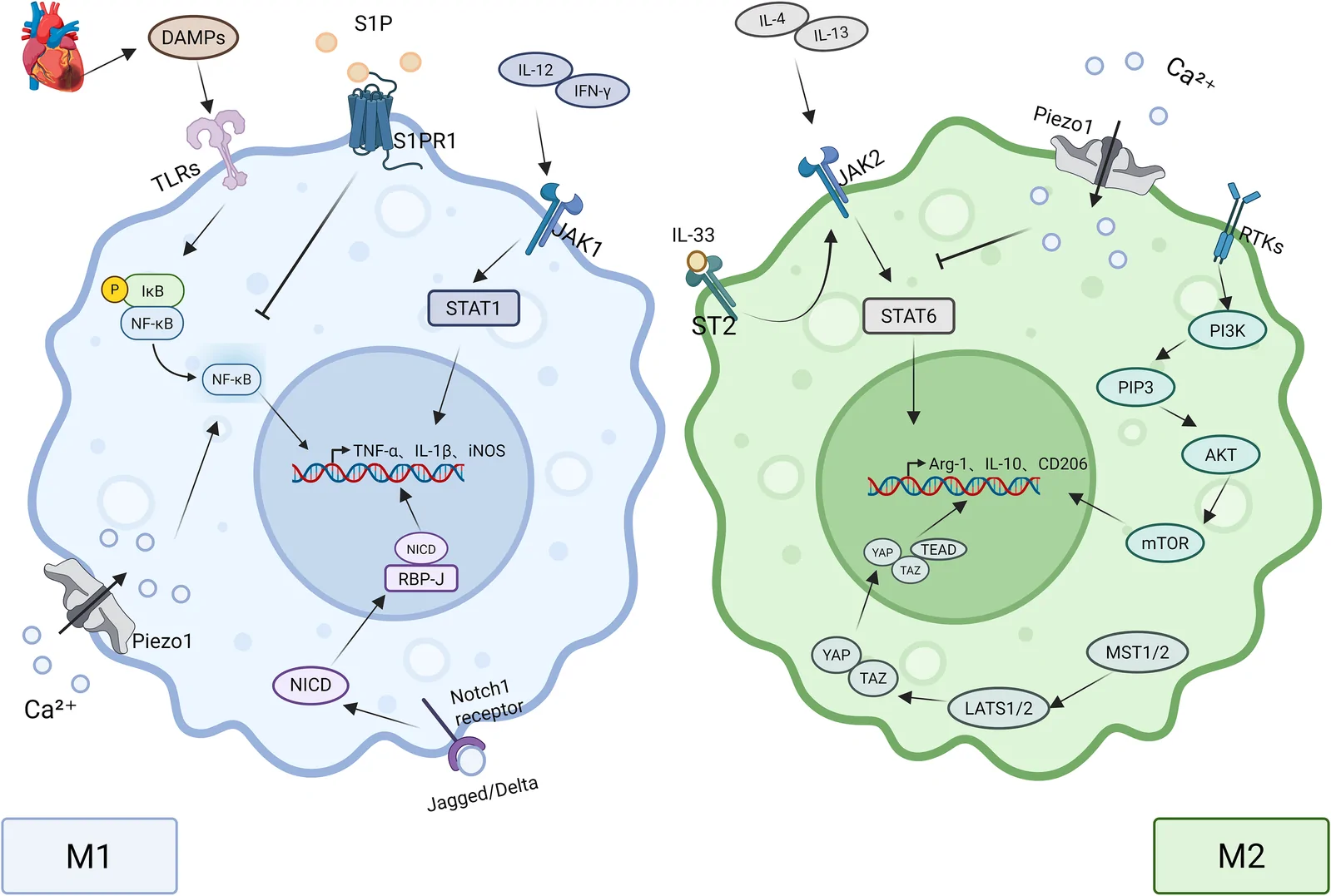
Key signaling pathways regulating macrophage polarization in AMI. Macrophages polarize into M1 or M2 types in response to cytokines and environmental signals. After myocardial infarction, necrotic myocardial cells release DAMPs, which activate the NF-κB pathway, driving M1 polarization and inducing the production of pro-inflammatory cytokines. Activation of the S1P/SK1/S1PR1 pathway can inhibit NF-κB, reducing the inflammatory response. The JAK-STAT pathway promotes M1 polarization through STAT1 activation (induced by IL-12 and IFN-γ) and M2 polarization through STAT6 activation (induced by IL-4, IL-13, and IL-33). The PI3K/Akt pathway supports cardiac repair by enhancing M2 polarization. The Hippo pathway regulates M2 polarization during tissue repair by modulating YAP/TAZ transcriptional activity. The Notch pathway maintains M1 activation through the NICD-RBP-J complex, promoting the expression of pro-inflammatory genes. Piezo1 influences macrophage polarization by sensing ECM stiffness. In a high-stiffness environment, Piezo1 activates pro-inflammatory pathways such as NF-κB, inhibits STAT6 activation, and reduces the expression of repair markers.

The NF-κB signaling pathway is a core regulator of macrophage polarization in AMI, responding to DAMPs released by necrotic myocardial cells by regulating the transcription of pro-inflammatory genes (75). When upstream receptors (such as Toll-like receptors (TLRs) and cytokine receptors) are activated, IKK (IκB kinase) phosphorylates IκBα, leading to its ubiquitination and proteasomal degradation (76). This process releases NF-κB subunits (mainly p65/p50), allowing them to translocate to the nucleus and induce the transcription of M1-associated genes such as TNF-α, IL-1β, and iNOS (21, 77) (**Figure 2**). In AMI, NF-κB activation occurs rapidly after ischemic injury, driving the early pro-inflammatory phase needed to clear necrotic tissue. However, sustained activation of NF-κB keeps macrophages in the M1 phenotype, further exacerbating tissue damage and hindering the transition to the repair-oriented M2 phenotype.

Therefore, regulating the activity of the NF-κB pathway may help promote the resolution of inflammation after AMI, balance macrophage polarization, and improve cardiac repair. For example, research shows that IL-34 activates the IKK complex, phosphorylates IκBα, and promotes its degradation, releasing the NF-κB p65 subunit, which translocates to the nucleus and activates the transcription of pro-inflammatory genes. This process promotes M1 macrophage polarization and enhances the inflammatory response after AMI, further exacerbating cardiac damage. Deletion of IL-34 significantly inhibits this pathway, reducing inflammation and cardiac damage (21); additionally, a study by Zhai et al. found that circ_0060745 expression was significantly elevated in the myocardium of AMI mice, particularly in cardiac fibroblasts. Silencing circ_0060745 significantly inhibited the activation of the NF-κB signaling pathway, reduced the expression of NF-κB p65, and lowered the p-IκBα/IκBα ratio. This, in turn, suppressed the production of pro-inflammatory cytokines. Furthermore, circ_0060745 knockdown also significantly inhibited macrophage migration under hypoxic conditions, thereby impairing the repair process following cardiac injury (78).

### 3.2 JAK-STAT signaling pathway

The JAK-STAT pathway plays a key role in macrophage polarization (79). This pathway is activated by cytokine receptors, which activate JAK family kinases, leading to phosphorylation of STAT transcription factors. This results in their dimerization, nuclear translocation, and transcriptional activation (80). Specifically, when cytokines such as IL-4 and IL-13 bind to receptors on macrophage surfaces, JAK2 kinase is activated and phosphorylates STAT6, promoting its translocation to the nucleus and initiating the expression of M2 macrophage characteristic genes, thus promoting anti-inflammatory and tissue repair functions (81, 82) (**Figure 2**). Conversely, IL-12 and IFN-γ promote M1 polarization by activating the JAK1/STAT1 pathway, enhancing the production of pro-inflammatory cytokines, and driving the inflammatory response (83). Therefore, the JAK-STAT pathway plays a balancing role between M1 and M2 macrophage polarization and significantly impacts cardiac repair and the inflammatory response after AMI.

It is important to recognize that after AMI, macrophage polarization is a complex process that is finely regulated by multiple signaling pathways. These pathways do not function in isolation but interact with one another, collectively shaping the immune environment and influencing cardiac repair and remodeling. The actions of these pathways often overlap and conflict, and their combined effects are not always straightforward or linear. Instead, the final macrophage function is shaped by dynamic interactions, feedback regulation, and adjustments across various stages. The NF-κB and JAK-STAT pathways are crucial in the early inflammatory response, but their roles are not always consistent. In the initial stages, these pathways cooperate to promote M1 polarization and amplify the inflammatory response. However, their contributions diverge significantly during the repair phase. As repair begins after AMI, IL-33 activates the JAK-STAT pathway, steering macrophages toward an M2 polarization, which reduces local inflammation and promotes tissue repair (84). In contrast, sustained activation of the NF-κB pathway may inhibit this transition, keeping macrophages in a pro-inflammatory state and impeding the repair process. Thus, the interaction between these two pathways is not merely “enhanced” or “antagonistic” but is modulated according to different spatiotemporal conditions, ensuring a rapid shift toward repair after inflammation resolution. If the interaction between these pathways is overlooked, it may lead to the erroneous assumption that the NF-κB and JAK-STAT pathways are in direct opposition to one another. This could result in underestimating their regulatory roles at different stages, potentially simplifying intervention strategies and failing to establish the optimal immune balance necessary for effective repair.

### 3.3 PI3K/Akt signaling pathway

In the PI3K/Akt signaling pathway, PI3K catalyzes the conversion of phosphatidylinositol (PIP2) to phosphatidylinositol-3-phosphate (PIP3) via its p110 subunit, which in turn activates Akt kinase and initiates a series of downstream signaling events (85). Upon activation, Akt can affect cell metabolism, proliferation, survival, and immune responses by inhibiting GSK3β (glycogen synthase kinase 3β), activating mTOR (mechanistic target of rapamycin), and regulating the activity of FOXO transcription factors (86, 87). During macrophage polarization, the PI3K/Akt pathway particularly promotes M2 macrophage polarization, enhancing its anti-inflammatory properties and promoting the expression of repair-related genes, such as Arg-1 and IL-10 (88, 89)(**Figure 2**). Conversely, inhibition of the PI3K/Akt signaling leads to M1 macrophage activation, which subsequently promotes the production of pro-inflammatory factors, thereby exacerbating the inflammatory response and worsening heart injury (90). Thus, the PI3K/Akt pathway plays a crucial role in balancing immune responses and promoting tissue repair by regulating macrophage polarization.

For example, Shuxuening injection (SXNI) has been shown to promote the transition of macrophages from M1 to M2 following AMI by modulating the PI3K/Akt signaling pathway (91). Studies indicate that after SXNI treatment, the phosphorylation levels of PI3K and Akt in heart tissue of MI rats significantly increased, suggesting the pivotal role of this pathway in macrophage polarization regulation. Through this mechanism, SXNI not only suppressed the expression of pro-inflammatory markers from M1 macrophages, but also promoted the expression of M2 markers, thereby alleviating the inflammatory response and improving cardiac function (91, 92).

### 3.4 Hippo signaling pathway

The Hippo signaling pathway primarily regulates cell proliferation and tissue homeostasis, and its role in macrophage polarization, particularly during inflammation, has garnered increasing attention. Central to the Hippo pathway is the kinase cascade (93), with its key mediators, YAP (Yes-associated protein) and TAZ (transcriptional co-activator with PDZ-binding motif), functioning as transcriptional regulators involved in cardiac regeneration and repair (94). Ste20-like kinases 1/2 (MST1/2) form complexes with Salvador family WW domain-containing proteins (SAV1), which phosphorylate and activate large tumor suppressors 1/2 (LATS1/2). LATS1/2 subsequently phosphorylate and inhibit YAP and TAZ, two transcriptional co-activators. Upon dephosphorylation, YAP/TAZ translocate to the nucleus, where they interact with various transcription factors such as TEAD to regulate gene expression (95)(**Figure 2**).

During the inflammatory phase of AMI, pro-inflammatory signals in the microenvironment activate the core kinases MST1/2 and LATS1/2 of the Hippo pathway. This leads to the phosphorylation of YAP/TAZ, causing them to remain in the cytoplasm and undergo degradation, thus suppressing the transcription of repair-related genes. This mechanism sustains macrophage polarization toward the pro-inflammatory M1 phenotype to clear necrotic tissue (96). Conversely, during the repair phase, increased tissue stiffness and signals like IL-4 inhibit LATS1/2 kinase activity, allowing YAP/TAZ to undergo dephosphorylation. This process enables their entry into the nucleus, where they bind to transcription factors such as TEAD and initiate the expression of M2-type marker genes like Arg1. Through cooperation with pathways such as STAT6, this promotes macrophage polarization to the anti-inflammatory M2 phenotype, facilitating collagen deposition and angiogenesis, and ultimately completing cardiac tissue repair (97, 98).

Notably, the absence of MST1/2 in macrophages leads to further deterioration of cardiac function after myocardial infarction. MST1/2-deficient mice exhibit significant cardiac dysfunction, including increased left ventricular volume and decreased ejection fraction, highlighting the critical role of MST1/2 in cardiac repair following infarction (99). Compared to wild-type mice, MST1/2-deficient mice show markedly impaired cardiac repair, underscoring the vital role of MST1/2 in the repair process (100).

Similarly, the roles of the PI3K/Akt and Hippo pathways in the repair process are not straightforward. During the cardiac repair phase, the PI3K/Akt pathway facilitates M2 macrophage polarization and supports tissue regeneration by promoting the secretion of anti-inflammatory and repair-associated factors. In contrast, the activation of the Hippo pathway during this phase generally inhibits the repair process by suppressing the activity of YAP/TAZ, which in turn restricts the expression of genes involved in repair. Although the PI3K/Akt pathway is beneficial for tissue repair, the concurrent activation of both pathways may lead to the inhibitory effects of the Hippo pathway undermining the repair-promoting actions of PI3K/Akt, resulting in a delayed M1-to-M2 transition. This phenomenon underscores the importance of pathway coordination and regulation in ensuring effective cardiac repair. Simply activating a single pathway may not be sufficient for optimal repair; rather, the interaction between these pathways is crucial for achieving the desired outcome.

### 3.5 Notch signaling pathway

The Notch signaling pathway plays a critical role in macrophage polarization post-AMI. Upon binding of the Notch1 receptor with its ligands, such as Jagged or Delta, the Notch signaling pathway activates its intracellular domain (NICD) (101). The NICD then enters the nucleus and binds to the transcription factor RBP-J to regulate the transcription of specific genes (102). Notch signaling regulates macrophage polarization, particularly by inducing the activation of M1 macrophages, which promotes the production of pro-inflammatory factors (103)(**Figure 2**). This M1 polarization helps clear necrotic tissue and combat pathogens, but prolonged activation of Notch signaling exacerbates inflammation and triggers tissue damage. Studies have shown that inhibiting Notch signaling promotes M2 macrophage polarization, alleviates cardiac inflammation, and accelerates the repair process.

For example, research by Li et al. demonstrated that blocking the Notch signaling pathway significantly improved cardiac injury in a myocardial infarction mouse model. Using an RBP-J inhibitor, macrophage polarization shifted toward M2, resulting in a significant reduction in pro-inflammatory factors, and also suppressed the expression of fibrosis-related factors, such as TGF-β1 and COL1. Additionally, the interaction of the Notch signaling pathway with other pathways exerts multiple regulatory effects. In particular, Notch inhibition reduces cardiac fibrosis by suppressing the activation of the NF-κB pathway (104). Thus, Notch not only plays a critical role in the transition between M1/M2 polarization but also modulates the balance between inflammation and repair at various stages of the cardiac repair process. This dynamic regulation highlights the importance of Notch signaling in maintaining the appropriate immune response throughout different phases of heart tissue remodeling. Yin et al. found that treatment with the Notch inhibitor DAPT significantly enhanced M2 macrophage activation, reduced levels of inflammatory factors, and markedly decreased the expression of nerve growth factor (NGF), thus slowing the overgrowth of cardiac sympathetic nerves and reducing the occurrence of fatal ventricular arrhythmias (105).

The interactions between these pathways highlight the complexity and dynamic nature of signaling in cardiac repair. While these pathways are critical at various stages, the simplified M1/M2 model does not fully capture the multifaceted regulatory roles of these signaling networks. Macrophage polarization into M1 and M2 phenotypes is not a mutually exclusive process; rather, multiple states can exist simultaneously and interconvert within the same microenvironment. Moreover, the functions of individual signaling pathways are not unidirectional; they create a highly adaptable regulatory network, shaped by cross-feedback, time-dependent regulation, and environmental changes. As such, future therapeutic strategies should focus on this interactive regulation, rather than simply inhibiting or activating a single pathway. A deeper understanding of the synergies and conflicts between these pathways will offer new insights for developing more effective interventions.

## 4 Cardiac microenvironment and its comprehensive regulation of macrophage polarization

### 4.1 Microenvironmental regulation of immune factor networks

After AMI, the immune microenvironment of the heart undergoes significant changes, with the immune factor network playing a complex and delicate regulatory role. Immune factors precisely regulate macrophage polarization through local accumulation and interaction at the site of cardiac injury, which in turn influences the inflammatory response, repair processes, and ultimately tissue remodeling. Recent studies have revealed that immune factors not only directly affect macrophage polarization but also regulate other cells in the local microenvironment, such as endothelial cells, fibroblasts, and T cells, which work synergistically in the cardiac repair process.

Immune factors regulate macrophage polarization through multiple mechanisms, playing a key role in immune responses and tissue repair. IL-4 and IL-13 bind to the IL-4Rα and IL-13Rα1 receptors on the macrophage surface, activating the JAK1 and JAK3 tyrosine kinases, which phosphorylate the STAT6 transcription factor. Once phosphorylated, STAT6 dimerizes and translocates to the nucleus, where it initiates the transcription of M2 polarization-associated genes, thereby regulating the immune response and promoting tissue repair (81). In contrast, IFN-γ binds to its receptor on the macrophage surface, activating JAK1 and JAK2, which in turn phosphorylate and activate STAT1. Activated STAT1 dimerizes and translocates to the nucleus, where it drives the transcription of pro-inflammatory genes associated with M1 macrophages, such as TNF-α and IL-1β. This amplifies the local inflammatory response, facilitating tissue clearance and repair, but may also exacerbate inflammation and delay the repair process (83). Immune factors not only influence macrophage phenotype directly, altering their functional state, but also fine-tune macrophage actions by regulating cytokine and secretion release in different immune environments. This precise regulatory balance is crucial for the success of the cardiac repair process.

Immune factors regulate macrophage polarization not only through individual mechanisms but also by interacting through various pathways, forming a complex immune factor network. For example, S1P (sphingosine-1-phosphate) plays a bridging role in the cardiac immune microenvironment (106). Activation of the S1P/SK1/S1PR1 pathway induces macrophage polarization toward the M2 phenotype, enhancing its reparative and anti-inflammatory functions, thus alleviating inflammation and fibrosis after AMI. Additionally, this pathway inhibits the NF-κB signaling pathway in macrophages, reducing the expression of pro-inflammatory factors and consequently decreasing the inflammatory response after AMI (107). Meanwhile, S1P also regulates endothelial cell function, promoting neovascularization to provide necessary blood supply for cardiac repair (108). Thus, the role of S1P in the cardiac immune microenvironment extends beyond macrophages to other cell types, forming a multi-layered immune regulatory network.

In recent years, new mechanisms of immune factors in cardiac repair post-AMI have been continuously discovered. For example, immune checkpoint molecules such as PD-1 and CTLA-4 have attracted increasing attention for their role in macrophage polarization (109–112). Studies have found that inhibition of PD-1 signaling enhances M2 macrophage polarization, thereby reducing inflammation and fibrosis in the heart (113). Moreover, it has been discovered that exosomes, as novel immune factor carriers, have gained increasing recognition for their role in cardiac repair following injury (114, 115). Exosomes carry various immune factors that regulate macrophage polarization and the immune microenvironment, providing new therapeutic strategies for cardiac repair (116). These innovative studies offer new therapeutic targets for cardiac repair and reveal the multi-dimensional regulatory role of the immune factor network in immune modulation and tissue repair following AMI. By precisely regulating these immune factors and their networks, we may develop more effective treatments to promote cardiac repair and functional recovery after AMI.

### 4.2 Mechanical stress and extracellular matrix regulation

During cardiac repair following AMI, mechanical stress and changes in the extracellular matrix (ECM) play a critical role in macrophage polarization and cardiac repair. After AMI, the injured area of the heart often experiences high tension, tissue stretching, and remodeling of ECM components (117). These mechanical changes not only directly affect the behavior of macrophages through physical forces but also regulate the polarization state of immune cells, further influencing the cardiac repair process. Particularly in the infarcted region, the local mechanical environment and ECM remodeling play a pivotal role in regulating macrophage function and immune response, thereby determining the progress and effectiveness of cardiac repair.

During the acute phase of AMI, the cardiac injury area undergoes drastic changes in mechanical stress, especially as the damaged tissue is subjected to a high-tension environment. Mechanical stress, transmitted through ECM, activates the integrin-FAK signaling pathway, which in turn affects downstream signaling pathways, such as YAP/TAZ and PI3K/Akt, regulating macrophage polarization (118). The ECM not only provides structural support for the tissue during cardiac repair but also interacts with macrophage surface receptors to modulate their polarization state. Specifically, ECM remodeling, particularly fibrosis and collagen deposition, is often accompanied by M1 macrophage activation. In a high-stiffness environment, ECM tends to promote M1 macrophage polarization, enhancing the inflammatory response, while in a low-stiffness environment, macrophages tend to polarize toward the M2 phenotype, supporting repair and anti-fibrotic actions (119, 120). Additionally, glycosaminoglycan components in the ECM, such as hyaluronic acid, bind to the CD44 receptor, thereby activating the RhoA/ROCK signaling pathway. This activation inhibits NF-κB signaling, reducing the pro-inflammatory response of M1 macrophages and promoting M2 polarization. Consequently, this shift enhances the repair response and exerts anti-fibrotic effects, further supporting tissue regeneration and mitigating fibrosis (121). ECM and glycopeptide hybrid hydrogels create a microenvironment similar to that of the natural ECM, which promotes M2 macrophage polarization and regulates the inflammatory cascade following myocardial infarction (122). The stiffness changes in ECM regulate macrophage immune phenotypes by altering receptor pairing and activating downstream signals, thereby affecting the fibrosis process and tissue repair outcome in the heart.

In this process, Piezo1, a key mechanosensitive ion channel, plays an important role. Studies have shown that Piezo1 regulates macrophage polarization by sensing changes in ECM stiffness (123). In a high-stiffness ECM environment, activation of Piezo1 induces Ca^2+^ influx, which activates pro-inflammatory signaling pathways such as NF-κB, thereby enhancing the inflammatory response of M1 macrophages (124). Additionally, the Ca^2+^ influx through the Piezo1 channel inhibits STAT6 activation and the expression of healing markers, thus suppressing the reparative functions of M2 macrophages (123). In contrast, in a low-stiffness ECM environment, the inhibition of Piezo1 facilitates M2 macrophage polarization, promoting repair and anti-fibrotic actions (120).

In conclusion, the complex interplay between mechanical stress, ECM stiffness, and macrophage polarization plays a central role in cardiac repair after AMI. By modulating the mechanical environment and related biomaterials, particularly the application of glycopeptide-hybrid hydrogels, it is possible to promote macrophage transformation toward the M2 phenotype, enhancing repair and anti-fibrotic responses. This provides new insights and potential therapeutic strategies for future clinical treatments of cardiac repair.

### 4.3 Synergistic interactions with other immune cells

During cardiac repair following AMI, macrophage polarization is influenced not only by local environmental factors but also by the synergistic interactions with other immune cells. Regulatory T cells (Tregs), dendritic cells (DCs), and CD8+ T cells play crucial roles in the heart’s immune response, regulating macrophage immune phenotypes and, to some extent, determining the inflammatory response and tissue repair outcomes during the repair process.

Treg cells, through direct interactions and exosome-mediated effects, synergistically regulate macrophage polarization, exerting immune-suppressive and reparative functions, thereby providing potential therapeutic strategies for post-AMI treatment. Treg cells interact directly with macrophages, effector T cells, and other immune cells to secrete inhibitory cytokines, directly suppressing pro-inflammatory responses, alleviating the inflammatory response after myocardial infarction, slowing cardiac remodeling, and protecting cardiomyocytes from apoptosis (125, 126). Moreover, exosomes derived from Treg cells can suppress pro-inflammatory factors from M1 macrophages, while upregulating anti-inflammatory factors from M2 macrophages. This helps reduce infarct size, inhibits cardiomyocyte apoptosis, and improves outcomes after AMI (116).

DCs, as important antigen-presenting cells, also play a pivotal role in immune regulation post-AMI. DC capture and process antigens, presenting them to T cells via MHC molecules to activate antigen-specific T cells. Through the secretion of cytokines such as IL-12 and TGF-β, DC cells regulate T cell polarization, promoting the differentiation of Th1 or Treg cells. In addition to modulating T cell immune responses, DC cells influence macrophage polarization, facilitating the transition from M1 to M2 macrophages, which reduces inflammation and promotes tissue repair. Choo et al. generated tolerogenic dendritic cells (tDCs) by treating mouse bone marrow-derived DCs with TNF-α and lysates from infarcted myocardium. After inducing AMI in C57BL/6 mice by ligating the left anterior descending coronary artery, they subcutaneously injected tDCs twice, once within 24 hours and again 7 days after surgery. Following injection, the tDCs migrated to the local lymph nodes, where they induced the activation and expansion of myocardial infarction-specific Tregs. These activated Tregs then entered the bloodstream and infiltrated the infarcted myocardium, promoting the conversion of macrophages from the M1 to M2 phenotype. This ultimately improved cardiac remodeling, enhanced left ventricular function, and increased the survival rate of the treated mice (127). Zhang et al. further showed that exosomes derived from DCs (DEXs) significantly upregulated the infiltration of Treg cells and M2 macrophages in the peri-infarct region, activating Treg cells and converting macrophages to reparative M2 macrophages, a process confirmed both *in vitro* and *in vivo* (128).

CD8+ T cells also play a complex role in the immune response following AMI. By directly killing infected or transformed cells, CD8+ T cells contribute to the clearance of necrotic myocardium. Activated CD8+ T cells release granzyme B, which has cytotoxic activity in the heart and promotes myocardial cell death and sterile pro-inflammatory immune responses (129). While early activation of CD8+ T cells helps eliminate infected cells (130), later in AMI, CD8+ T cell activation may be closely linked to the activation of M1 macrophages, thereby intensifying local inflammatory responses (131–133). It has been shown that 21 days after myocardial infarction, depletion of systemic CD8+ T cells can explain the shift in macrophage phenotype to an anti-inflammatory state, significantly reducing ischemic heart damage and improving cardiac function following AMI (129). Therefore, the removal of CD8+ T cells can significantly decrease myocardial cell apoptosis, myocardial inflammation, and infarct size, ultimately improving myocardial function. However, the function of CD8+ T cells is also regulated by Treg cells. Treg cells suppress excessive activation of CD8+ T cells by secreting immunosuppressive factors, helping maintain the balance of the immune response (134). Therefore, the balance and interaction between CD8+ T cells, Treg cells, and DCs influence macrophage polarization and, to some extent, determine the outcome of cardiac repair.

The synergistic interactions of these immune cells form a complex immune regulatory network that plays a crucial role in the cardiac repair process following AMI. Through the interactions between Treg cells, DCs, CD8+ T cells, and other immune cells, the polarization state of macrophages is finely regulated, influencing the degree of inflammation and the efficiency of tissue repair. More broadly, cardiac repair depends not only on the action of these immune cells but also on the joint regulation by other immune cells and molecules (22). The complex synergy between immune cells determines the ultimate outcome of the immune response after AMI, providing strong immune support for cardiac repair.

### 4.4 Modulatory role of individual immune background

Individual immune background, particularly differences in sex and age, plays a significant role in the immune response following AMI, especially in the timing and characteristics of macrophage polarization. Understanding how factors such as sex and age influence macrophage polarization helps reveal its key role in cardiac repair and provides a theoretical basis for the development of personalized treatment strategies.

Sex differences have a significant impact on immune responses, particularly during AMI and cardiac repair (135). There are distinct differences in AMI incidence, disease progression, and immune responses between men and women. Specifically, men typically have a higher incidence of AMI in their middle-aged years, while women experience a sharp increase in cardiovascular disease risk after menopause, with their incidence gradually approaching that of men (136, 137). Although the specific mechanisms behind sex differences remain incompletely understood, sex hormones play a crucial role in regulating these differences. Testosterone levels are higher in men, while women have higher levels of estrogen prior to menopause. These sex hormones not only regulate reproductive functions but also exert significant modulatory effects on the immune system (138, 139). For instance, research indicates that 17β-estradiol (E2) increases the proportion of M2 macrophages, reduces the activation of M1 macrophages, suppresses the inflammatory response, and promotes repair, improving outcomes after AMI. E2 activates antioxidant pathways, inhibits superoxide generation, and modulates macrophage polarization by increasing repair factors, while reducing pro-inflammatory factors (140). This action of estrogen helps alleviate cardiac inflammation and fibrosis, promoting cardiac repair, and offers a new direction for treating AMI, especially in postmenopausal women, where the decline in estrogen levels increases cardiovascular disease risk. Therefore, interventions targeting this mechanism may provide new treatment strategies (141, 142). As research into these mechanisms progresses, future clinical interventions may improve cardiac repair after AMI by modulating estrogen levels or mimicking estrogen’s effects.

The risk and prevalence of AMI gradually increase with age (143). In elderly AMI patients, macrophage polarization undergoes significant changes, profoundly impacting immune responses and the cardiac repair process. Compared to younger AMI patients, elderly patients tend to have a polarization of macrophages toward the pro-inflammatory M1 phenotype, with a lower proportion of reparative M2 macrophages (19). Studies have shown that in elderly AMI patients, the activation of M1 macrophages is more pronounced, accompanied by high levels of pro-inflammatory cytokines, which may exacerbate local inflammation and fibrosis (144). Additionally, left ventricular (LV) free wall rupture is one of the deadliest complications of AMI, accounting for 5-31% of all in-hospital mortality. Observational studies have found a higher incidence of LV free wall rupture in elderly patients with first-time AMI, indicating that advanced age is an important risk factor for cardiac rupture after AMI (145). Therefore, age significantly influences macrophage polarization, leading to stronger pro-inflammatory responses, which in turn exacerbates delayed cardiac repair and fibrosis. Treatment strategies targeting macrophage polarization imbalance may become an effective approach to improve the prognosis of elderly AMI patients.

The differences in sex and age play an undeniable role in the immune response following AMI. Sex factors regulate macrophage polarization through sex hormones, particularly enhancing the repair response by promoting M2 polarization via estrogen. As individuals age, changes in macrophage polarization lead to delayed repair and heightened inflammation in elderly patients. Therefore, future treatment strategies should take into account factors such as sex and age, incorporating individual immune backgrounds to further optimize the regulation of macrophage polarization and provide more precise therapeutic approaches for cardiac repair after AMI.

## 5 Macrophage polarization intervention strategies and clinical research progress

Macrophage polarization plays a crucial role in immune response and cardiac repair following AMI. By regulating macrophage polarization, particularly the balance between M1 and M2 phenotypes, it is possible to significantly improve cardiac repair and remodeling. In recent years, with the deepening understanding of macrophage polarization mechanisms, a growing number of intervention strategies have emerged, including pharmacological interventions, gene editing, cell therapy, and nanomedicine delivery systems (**Table 1**). These strategies aim to regulate macrophage polarization to promote cardiac repair and alleviate inflammatory responses. These approaches not only help modulate the polarization direction of macrophages but also have the potential to provide new breakthroughs in clinical treatment.

**Table 1:** The relevant drugs for different intervention strategies in recent years.

Intervention strategies	Drugs	Mechanism	References
Pharmacological and Molecular Interventions Targeting Polarization Pathways	SAA1 inhibitors	SAA1 promotes M1 macrophage polarization by activating the p38 MAPK signaling pathway, leading to exacerbated inflammation and aggravated cardiac injury. In contrast, the absence of SAA1 inhibits M1 macrophage polarization and facilitates the conversion of M1 to M2 macrophages, thereby alleviating the inflammatory response and reducing cardiac damage.	(185)
	Dichloroacetate(DCA)	DCA significantly improved cardiac function in MI mice, as evidenced by reduced myocardial injury and lower levels of CK-MB and LDH. It also decreased the levels of inflammatory cytokines and promoted the polarization of macrophages from M1 to M2.	(146)
	Nitro-oleic acid(OA-NO 2 )	OA-NO2 alleviates myocardial fibrosis and the inflammatory response by inhibiting the activation of cardiac fibroblasts and the polarization of M1 macrophages, thereby improving cardiac function after AMI.	(186)
	GLP-1/GLP-2 receptor dual agonist	EAT promotes M1 macrophage polarization, inducing MVO formation and worsening myocardial injury after AMI. Therapeutic strategies targeting EAT, such as GLP-1/GLP-2 receptor dual agonists, may provide a new approach for improving cardiac function recovery following AMI.	(187)
	a protein inhibitor of galectin-3 (Gal-3C)	Galectin-3 promotes macrophage polarization toward the M2 phenotype, aiding in cardiac repair, but it may also contribute to cardiac fibrosis. Studies have shown that inhibiting Galectin-3, such as with the Gal-3C inhibitor, can reduce cardiac fibrosis and improve outcomes after AMI.	(188)
	IL-34	IL-34 activates the NF-κB signaling pathway, promoting the recruitment and polarization of CCR2+ macrophages. It enhances the function of anti-inflammatory M2 macrophages, thereby alleviating ischemia-reperfusion injury after AMI.	(21)
Gene/Cell Therapy	ADSC-EXOs	ADSC-EXOs, through the bioactive molecules they contain, promote the polarization of macrophages toward the M2 phenotype, exert anti-inflammatory effects, alleviate the inflammatory response, and promote tissue repair.	(115)
	The protein IL-4-induced gene 1 (IL4I1)	IL4I1 regulates macrophage programming by influencing the phosphorylation of transcriptional activators and signaling factors (STAT3 and STAT6), which are involved in the polarization of macrophages toward M1 and M2 phenotypes, thereby alleviating AMI.	(157)
	Myeloid-derived suppressor cells (MDSCs)	MDSCs reduce the expression of pro-inflammatory cytokines and increase the levels of anti-inflammatory cytokines. They also reduce the infiltration of CD3+ T cells into the infarcted heart, promote M2 macrophage polarization, and inhibit inflammation. These actions contribute to the recovery of cardiac function and the alleviation of adverse cardiac remodeling after AMI.	(189)
	KLF4-induced upregulation of SOCS1	KLF4 alleviates H/R-induced AC16 cardiomyocyte damage by upregulating SOCS1 and enhances M2 macrophage polarization, providing a new strategy for the treatment of AMI.	(190)
	MSC-exo	By upregulating miR-125a-5p and inhibiting the TRAF6/IRF5 signaling pathway, macrophage polarization toward the M2 phenotype is promoted, reducing the inflammatory response after myocardial infarction, improving cardiac function and structure, and alleviating AMI.	(114)
	MSC^ATV^ -EV	MSC^ATV^-EV promotes M2 polarization via the miR-139-3p/STAT1 axis, thereby facilitating post-AMI cardiac repair and showing strong promise for clinical translation.	(27)
Nanomedicine Delivery Systems	GelMA/GelNB hydrogel patch	By promoting M2 macrophage polarization, independent of cellular or exogenous factors, the inflammatory response and fibrosis after myocardial infarction are alleviated, leading to improved cardiac function.	(191)
	poly(amino acid) hydrogel	By scavenging ROS, inhibiting cardiomyocyte apoptosis, and shifting macrophage polarization from pro-inflammatory M1 to anti-inflammatory M2, the inflammatory response is reduced, and angiogenesis is promoted, significantly improving ischemia-reperfusion injury after AMI.	(170)
	HM4oRL	During the AMI phase, HM4oRL treatment significantly inhibited the activity of pro-inflammatory macrophages in the damaged myocardium. HM4oRL activates the AMPK signaling pathway, helping to establish a microenvironment conducive to the activity of reparative macrophages, thereby reducing inflammation and promoting myocardial repair.	(192)
	legumain-guided ferulate-peptide nanofibers (LFPN)	LFPN’s multifunctional actions were validated in cellular assays and MI mouse models. Compared with FA alone, LFPN reprogrammed macrophage polarization, attenuated inflammatory responses, and induced endothelial neovascularization. Promoting angiogenesis while reshaping the inflammatory microenvironment constitutes an effective therapeutic strategy for AMI.	(193)
	MPGC4	MPGC4, composed of polyethylene glycol (PEG) hydrogel and composite gene nanocarriers (CTL4), promotes the polarization of pro-inflammatory M1 macrophages toward anti-inflammatory M2 macrophages and enhances cardiomyocyte survival. In a rat AMI model, MPGC4 targets CTL4 precisely to the infarcted region by clearing MMPs, reducing early inflammatory responses, promoting neovascularization, and improving the immune microenvironment after AMI.	(194)
	NIL10	NIL10 is a novel IL-10 receptor-targeted nanoparticle that regulates macrophage polarization, promoting the accumulation of M2 macrophages, thereby alleviating inflammation and fibrosis after AMI and improving cardiac function. Its mechanism of action involves the activation of the IL-10 receptor/STAT3 signaling pathway, providing a new strategy for the treatment of AMI.	(195)
	ColCaNPs	ColCaNP treatment significantly reduced the myocardial infarction area by approximately 45%, alleviated myocardial fibrosis, and lowered serum levels of CRP, TNF-α, and IL-1β. It also regulated M1/M2 macrophage polarization, inhibited the TLR4/NFκB/NLRP3 signaling pathway, and significantly suppressed pyroptosis and inflammation, thereby mitigating AMI-induced damage.	(168)
Modulatory Role of Traditional Chinese Medicine in Macrophage Polarization	Quercetin	By regulating the autophagy pathway, reducing the binding of Bcl-2 to Beclin-1, and enhancing M2 macrophage polarization, it exerts anti-inflammatory and cardioprotective effects in AMI.	(196)
	Panaxatriol saponin (PTS)	PTS directly binds macrophage STING and selectively suppresses the M1 STING–TBK1–IRF3/NF-κB axis, thereby reducing pro-inflammatory outputs with minimal impact on M2. Consequently, it attenuates inflammatory cell infiltration and paracrine-mediated injury within the infarct zone, ameliorating AMI pathology.	(197)
	Qishen granule (QSG)	QSG promotes the transition of macrophages from the M1 to M2 phenotype, inhibits the activation of the NLRP3 inflammasome, and subsequently suppresses the activation of the P2X7R-NEK7-NLRP3 pathway. This reduces the release of pro-inflammatory factors, alleviates myocardial ischemia/reperfusion injury, and improves cardiac function and repair after AMI.	(198)
	Shuangxinfang (PCF)	PCF reduces inflammation by inhibiting the activation of macrophages and regulating their polarization state after AMI. Meanwhile, the dual-heart formula alleviates myocardial injury, cell apoptosis, and fibrosis by suppressing the S100A9 and TLR4/NF-κB pathways, promoting cardiac repair, and ultimately improving cardiac function.	(199)
	Curcumin	Curcumin inhibits the secretion of IL-18 by macrophages, thereby alleviating the activation of the IL-18-TGFβ1-p-SMAD2/3 signaling pathway and reducing cardiac fibrosis. It also suppresses the activation of M1 macrophages, promotes the function of M2 macrophages, and improves cardiac repair while reducing fibrosis, ultimately enhancing cardiac function after AMI.	(200)

### 5.1 Pharmacological and molecular interventions targeting polarization pathways

M1 macrophages are closely associated with inflammation, tissue damage, and poor cardiac repair. Therefore, inhibiting M1 macrophage activation or converting them into M2 macrophages is an effective strategy for preventing myocardial injury after AMI. Pharmacological interventions targeting M1 polarization mainly focus on inhibiting its core pathways, such as NF-κB and JAK/STAT.

The NF-κB signaling pathway is one of the key pathways in M1 macrophage polarization, where pro-inflammatory cytokines are produced in large quantities, exacerbating cardiac inflammation. Therefore, NF-κB pathway inhibitors, such as Dichloroacetate, effectively reduce the levels of inflammatory cytokines and promote the polarization of macrophages from M1 to M2, thereby improving AMI (146). Furthermore, overexpression of PCSK9 in the myocardium and macrophages enhances M1 macrophage polarization by upregulating TLR4 and its downstream MyD88/NF-κB signaling pathway. Thus, PCSK9 inhibitors can inhibit the NF-κB signaling pathway and induce the shift of macrophages from the M1 to M2 phenotype, promoting myocardial repair after infarction (77, 147). Although PCSK9 inhibitors, such as Evolocumab and Alirocumab, are primarily used for lipid-lowering therapy, their potential role in regulating inflammatory responses and macrophage polarization, particularly in the context of cardiac repair, has been increasingly studied and confirmed. As a result, these inhibitors present a feasible strategy for inhibiting the NF-κB pathway and improving AMI. At the same time, the JAK/STAT signaling pathway, particularly STAT1 activation, is also closely related to M1 polarization. Inhibition of the JAK/STAT pathway, such as with Interleukin 33 (84), significantly suppresses the pro-inflammatory actions of M1 macrophages and increases the number of M2 macrophages, which not only reduces inflammation after AMI but also promotes cardiac repair. Activation of signaling pathways such as PPARγ is considered a key mechanism for inducing M2 macrophage polarization. PPARγ agonists, such as rosiglitazone and pioglitazone, effectively induce M2 polarization, promoting inflammation resolution and tissue repair (148, 149).

As research into macrophage metabolic reprogramming advances, targeting macrophage metabolic pathways has emerged as a promising new intervention strategy. M1 macrophages primarily rely on glycolysis, while M2 macrophages predominantly use fatty acid oxidation and the tricarboxylic acid cycle. Therefore, manipulating macrophage metabolism—whether by inhibiting critical enzymes in glycolysis, like LDHA, or promoting fatty acid oxidation—could be an effective way to modulate macrophage polarization and improve outcomes in myocardial infarction. Lactate dehydrogenase A (LDHA) is a metabolic enzyme that catalyzes the conversion of pyruvate to lactate in the glycolytic pathway (150). Chen et al. found that lactate production induced by LDHA promotes M2 macrophage polarization, creating a beneficial environment for cardiac regeneration. These findings suggest that targeting LDHA-mediated metabolic reprogramming to reduce ROS levels and induce M2 macrophage polarization could serve as a therapeutic target to enhance cardiac repair after myocardial infarction (151). However, because these metabolic pathways are interconnected with various cellular processes, simple metabolic interventions may influence other cell functions. Therefore, developing highly specific strategies to regulate the activity of individual enzymes—rather than broad interventions—may help minimize interference with other cellular systems, ultimately improving both the efficacy and safety of such treatments.

In clinical practice, drug interventions targeting polarization pathways are among the most developed treatment approaches, primarily by modulating the balance between M1 and M2 macrophages to mitigate inflammation and promote cardiac repair. Drugs targeting key pathways such as NF-κB and JAK/STAT can effectively inhibit M1 macrophage activation and encourage its conversion to M2 macrophages, thereby enhancing the immune response and repair processes following myocardial infarction. However, these therapies face several challenges. One significant issue is the poor selectivity and targeting of the drugs, which may lead to systemic side effects, particularly widespread impacts on the immune system (152). Additionally, the long-term effects of these drugs and their efficacy in diverse immune contexts have not been fully established, and individual variations, such as genetic factors, may contribute to inconsistent therapeutic outcomes (153). For example, a common and severe side effect of DCA is reversible peripheral neuropathy, caused by thiamine depletion and oxalate accumulation, leading to neurotoxicity. Moreover, DCA metabolism is influenced by age and GSTZ1 genotype, with children exhibiting a higher clearance rate compared to adults (154). In the translational process, the precision and tolerance of drug delivery systems need further refinement to ensure that the drugs are efficiently and accurately targeted to the intended sites.

Despite these challenges, drug interventions targeting polarization pathways remain a preferred option for treating macrophage polarization disorders due to their strong operability and broad clinical application, particularly in early-stage treatment. To enhance therapeutic efficacy, these drugs are often combined with other treatments. For example, gene editing technologies or exosome therapies can be employed to improve the targeting and efficacy of the drugs. By combining different therapeutic strategies, macrophage polarization can be more precisely controlled, thereby improving the overall treatment outcome. As such, the combination of drug interventions with gene therapy, cell therapy, and other approaches holds the potential to provide more comprehensive and personalized treatment options in future clinical applications.

### 5.2 Gene/cell therapy

Gene and cell therapies have become one of the key directions in current research to regulate macrophage polarization. By using techniques such as gene editing and exosome transfection, researchers aim to precisely control the functional state of macrophages, thereby optimizing myocardial repair and reducing inflammation following AMI. With advancements in technology, these therapeutic methods have gradually emerged as promising clinical treatments.

Gene editing technologies, especially the CRISPR-Cas9 system, have made significant progress in various fields. In the regulation of macrophage polarization, gene editing enables precise modification of target gene expression, thereby controlling macrophage function. For instance, by editing key factors that affect M1 and M2 polarization, such as IL4I1, IRAK-M, and HIMF, researchers can induce macrophages to shift toward specific polarization states, effectively suppressing inflammation or promoting tissue repair (155–157). In AMI models, the use of the CRISPR-Cas9 system to knock out or knock in specific genes can significantly improve the polarization process of macrophages, promote the proliferation and functional activation of M2 macrophages, and accelerate myocardial repair and recovery (158).

Exosomes, as intercellular signaling molecules, have been widely used in gene and cell therapies in recent years. Exosomes carry various bioactive molecules, such as miRNA, proteins, and lipids, and participate in intercellular material exchange and information transfer (159).In the regulation of macrophage polarization, researchers have effectively modulated macrophage polarization by transfecting exosomes carrying specific miRNAs or therapeutic molecules. Through exosome-mediated targeted delivery, therapeutic molecules can directly act on macrophages, allowing for precise regulation. Through exosome-mediated targeted delivery, therapeutic molecules are initially carried by exosomes and enter macrophages via specific binding with surface receptors. After fusion with the cell membrane, the therapeutic molecules are released into the cell, allowing for precise regulation of macrophage functions, such as modulating immune responses or promoting cell polarization. This process ensures that therapeutic molecules act precisely within the target cells (114, 116). For example, the transfer of miR-139-3p from MSC ATV-EVs to macrophages targets and inhibits specific signaling pathways, including the expression and activation of Stat1, thereby promoting M2 macrophage polarization and significantly enhancing cardiac repair after AMI (27). Compared to traditional drug delivery systems, this method offers higher targeting specificity and lower toxicity, enabling cardiac tissue repair without causing systemic side effects (160).

Despite the substantial clinical potential of gene editing and exosome therapy, these methods still face notable challenges. Gene editing technology allows for precise regulation of macrophage polarization, but its clinical application is hindered by limitations such as delivery efficiency, off-target effects, and long-term safety concerns (161, 162). Although exosomes provide a targeted delivery system with high precision and low toxicity, challenges persist in their large-scale production and quality control. Additionally, issues related to exosome stability and immunogenicity need further resolution (163).

Moreover, as our understanding of immune system mechanisms deepens, the combination of gene and cell therapies has become an emerging trend. By integrating gene editing with cell therapy, the macrophage polarization process can be more finely regulated. For instance, using gene editing techniques to knock out or activate specific genes, alongside the application of bone marrow-derived stem cells or exosome delivery systems, can enhance therapeutic outcomes (164, 165). In clinical practice, this combination therapy not only improves the effectiveness of individual treatments but also enables multi-level immune modulation, resulting in more efficient cardiac repair after AMI.

### 5.3 Nanomedicine delivery systems

Nanomedicine delivery systems have emerged as a critical frontier in regulating macrophage polarization. Compared to traditional drug delivery, nanotechnology allows for precise targeting of specific cardiac tissues and cell subpopulations through size effects, surface modifications, and targeted designs, thereby enhancing therapeutic efficacy and minimizing systemic side effects. In the inflammatory and repair processes following AMI, this precise drug delivery method offers new possibilities for immune modulation and tissue regeneration.

Nanoparticles can achieve selective targeting of macrophage subpopulations through surface modifications. Studies have found that macrophages in different polarization states express distinct surface receptors; for example, M1 macrophages express higher levels of scavenger receptors, while M2 macrophages express mannose receptors such as CD206 (166). By modifying nanoparticles with specific ligands or antibodies on their surface, drugs can be more efficiently delivered to the target subpopulation, thus enabling precise regulation of macrophage polarization direction (167). For instance, nanoparticles loaded with PPARγ agonists can promote M2 polarization and accelerate cardiac repair (148); nanoparticles containing colchicine can modulate M1/M2 macrophage polarization and inhibit the TLR4/NFκB/NLRP3 signaling pathways, reducing inflammation (168).

Hydrogels, another class of delivery platforms, have also shown advantages in localized drug release for the heart. Hydrogels have excellent biocompatibility and tunable degradation rates, enabling the formation of a local drug reservoir in the infarcted area, allowing for sustained release of immune-modulating factors (169). For example, exosomes or anti-inflammatory factors encapsulated in injectable hydrogels can create a sustained release effect at the myocardial site, maintaining the dominance of M2 macrophages, thereby improving cardiac remodeling (170). Compared to systemic administration, these hydrogel systems significantly enhance drug utilization efficiency and reduce side effects in non-cardiac tissues.

Nanotechnology can also serve as a multifunctional platform, integrating drug delivery with imaging monitoring to achieve combined diagnosis and treatment (171). Systems loaded with magnetic nanoparticles target the specific CD163 receptors on macrophage surfaces, precisely delivering imaging probes into the cells. MRI scans are then used to monitor the polarization status of macrophages in real-time. CD163, a marker receptor for M2 macrophages, is recognized, and the magnetic properties of the nanoparticles generate strong MRI signals (172). By dynamically tracking the distribution and accumulation of these nanoparticles, real-time changes in the ratio of M1 to M2 macrophages can be distinguished, reflecting the macrophage polarization process (167, 173). This method allows for monitoring dynamic changes in macrophage polarization and evaluating the regulatory effects of immunomodulatory drugs on macrophage function, providing precise therapeutic feedback. This “visualized immunotherapy” strategy offers a promising direction for clinical translation. Additionally, nanomedicine delivery systems have the potential to be combined with other therapeutic modalities. By integrating with gene therapy or exosome delivery, nanoplatforms can serve as carriers to achieve more stable and targeted delivery of miRNAs, thus regulating the macrophage transcriptional network. For instance, using functionalized mesoporous silica nanoparticles (MSNs) to deliver microRNA-21-5p to the infarcted area may provide a new strategy to suppress M1 polarization, reduce inflammation, and promote local angiogenesis, thereby accelerating cardiac repair (174).

Despite the promising potential of nanodrug delivery systems, their clinical translation faces several significant challenges. Although these systems can enhance drug targeting, issues such as stability, immunogenicity, and *in vivo* pharmacokinetics still require optimization. The biocompatibility and degradability of nanoparticles are key factors that influence their long-term safety in the body, which remains one of the primary barriers to their clinical application (175). Furthermore, the production and standardization of nanodrug delivery systems are complex, particularly in ensuring consistent nanoparticle properties and precise drug loading capacity (176). While nanodrug delivery systems have shown substantial advantages in experimental settings, overcoming these technical hurdles is essential for their successful transition to large-scale clinical use.

In conclusion, nanomedicine delivery systems offer novel approaches for regulating macrophage polarization, with core advantages in precision, efficiency, and controllability. Future research needs to address issues such as the long-term safety of nanomaterials, *in vivo* pharmacokinetics, and clinical-scale preparation. Nevertheless, the development of this field will undoubtedly open new paths for immune intervention and cardiac repair following AMI.

### 5.4 Modulatory role of traditional Chinese medicine in macrophage polarization

Traditional Chinese medicine (TCM) primarily derives from plants, animals, and minerals, which undergo specific processing and preparation methods to enhance their therapeutic effects and reduce toxicity. TCM is usually administered in the form of decoctions, powders, capsules, or tablets. The various herbs in a formula work together to exert therapeutic effects. In regulating macrophage polarization, TCM’s role is not only seen in its direct pharmacological effects but also in its holistic approach, mild treatment methods, and personalized treatment plans. The personalized treatment aspect of TCM is based on the principle of “differentiated diagnosis and treatment,” where physicians adjust the combination and dosage of herbs based on a patient’s specific symptoms and constitution. This approach focuses on tailoring the treatment to the individual to achieve the best possible therapeutic outcomes. Compared to traditional single-drug treatments, TCM better meets the patient’s needs through the synergistic action of multiple herbs and personalized dosage adjustments, reflecting its personalized treatment advantages. More importantly, TCM has been used in clinical practice for thousands of years (57).

In contrast to the single-target treatment approach of Western medicine, TCM coordinates immune system responses by modulating multiple immune pathways and targets (177). In the process of macrophage polarization, certain Chinese herbal medicines can promote M2 macrophage polarization by regulating immune factor expression and alleviating inflammation induced by M1 macrophages. For example, Nuanxinkang (NXK) inhibits M1 macrophage polarization, reduces inflammation, and promotes M2 macrophage polarization, enhancing anti-inflammatory effects. In a pig model of myocardial infarction, NXK improves macrophage function by modulating the HIF-1α/PDK1 axis, promoting the conversion of macrophages to the M2 phenotype, and thus accelerating cardiac repair. This multi-pathway immune modulation helps promote cardiac regeneration (36). Additionally, the mild regulatory characteristics of TCM prevent excessive suppression of inflammation, avoiding complications such as infections due to excessive immune suppression. TCM often adopts the principle of “syndrome differentiation and treatment,” providing personalized treatment based on an individual’s constitution and pathological status, thereby precisely regulating macrophage polarization to achieve the optimal balance of immune response and cardiac repair.

In TCM, the theory of Yin-Yang balance serves as a core framework for understanding both the natural world and human health. Many physiological phenomena and pathological changes can be interpreted through this lens. The polarization of M1 and M2 macrophages can also be understood within this framework. Yin and Yang constrain each other: M1 macrophages drive pathological progression by intensifying inflammation and tissue damage, while M2 macrophages mitigate damage by reducing inflammation and promoting tissue repair. Together, they maintain the Yin-Yang balance of the body, ensuring normal physiological functions. Under certain conditions, M1 and M2 macrophages can transform into one another, which aligns with the TCM concept of Yin-Yang transformation. Regulating macrophage polarization aims to maintain the balance between M1 and M2, which mirrors the TCM approach of balancing Yin and Yang (178).

Although TCM is not traditionally used for treating cardiovascular diseases in Western medicine, an increasing body of research shows that it has significant effects in lowering blood pressure, improving cholesterol levels, and reducing inflammation (179–181). As TCM research deepens, particularly in uncovering the mechanisms of herbal components in macrophage polarization, its application in immune regulation, macrophage polarization, and cardiac repair is becoming increasingly promising. Therefore, further research is essential to better understand the potential of TCM in treating cardiovascular diseases. Moreover, the integration of modern medicine and TCM opens up new possibilities for treating diseases such as AMI and provides unique perspectives for precision medicine and personalized treatment.

Despite the unique advantages of TCM in regulating macrophage polarization, its clinical application still faces several significant challenges. The mechanisms of action of TCM are complex, involving the combined regulation of various bioactive components and immune pathways. However, the lack of clear molecular mechanism studies and systematic clinical validation has made the standardization and dosage control of TCM treatments challenging in modern medicine (182). Additionally, the quality and composition of different herbs and prescriptions are inconsistent, which leads to variability in treatment outcomes and complicates individualized therapy. While TCM has been used for thousands of years, there is limited foundational research in its modernization and clinical trials. The absence of large-scale, randomized controlled trials to support its widespread use restricts its effectiveness and general applicability in contemporary clinical practice (183). To address these issues, the combined use of TCM and modern therapeutic approaches is emerging as a potential solution. Modern drug therapies, while effective, can sometimes cause immune imbalances or excessive suppression, leading to side effects such as immunosuppression and infections. TCM, with its holistic approach, can mitigate these side effects by promoting immune balance and enhancing immune function (184). For example, after the administration of immunosuppressive drugs or chemotherapy, traditional herbs like Astragalus and Codonopsis can boost the immune system, regulate macrophage polarization, restore immune function, and accelerate tissue repair. This combination therapy not only enhances the safety of treatment but also improves its effectiveness while minimizing side effects.

### 5.5 Challenges and considerations in macrophage polarization intervention strategies

Macrophage polarization-based therapeutic interventions are a promising strategy for improving cardiac repair after AMI. These interventions primarily aim to regulate the balance between pro-inflammatory M1 macrophages and reparative M2 macrophages to promote myocardial healing. However, despite their great potential, we must carefully assess the broader effects of these strategies, especially considering the pivotal role macrophages play in systemic immunity.

Macrophages are crucial not only in cardiac tissue repair but also in the overall immune response. Therefore, therapies designed to enhance M2 polarization or suppress M1 activity may unintentionally affect other organs and systems. For example, shifting macrophage polarization toward the anti-inflammatory M2 phenotype could impair the immune system’s ability to effectively combat infections or eliminate pathogens, which is particularly concerning for patients with weakened immune systems or those prone to infections. Systemic modulation of macrophage polarization might disrupt immune homeostasis, leading to immunosuppression or immune imbalance. Prolonged suppression of M1 macrophages—essential for fighting infections and initiating inflammation—could make individuals more vulnerable to bacterial or viral infections. Conversely, excessive or uncontrolled M1 polarization could intensify chronic inflammation and contribute to the onset of other inflammatory diseases.

Furthermore, while gene editing technologies and nanomedicine delivery systems that specifically target macrophage polarization pathways offer precise regulation of macrophage activity, they also raise concerns about off-target effects and long-term safety. These technologies, though promising, may impact other cellular functions or cause unintended genetic changes, highlighting the need for careful consideration in future research. Additionally, the long-term effects of these therapies, including the potential for immune tolerance or resistance, must be further investigated.

In conclusion, while macrophage polarization-based therapies offer significant clinical potential in enhancing cardiac repair after AMI, their impact on the systemic immune system must be carefully evaluated. Future research should focus on balancing the therapeutic benefits with the preservation of systemic immune stability, ensuring that local repair is promoted without compromising overall immune function. This approach will help provide safer and more effective treatment options for patients.

Each of the four treatment strategies discussed above has its own advantages, yet they each face distinct challenges in clinical application. A comparison of these strategies reveals key insights. Drug interventions targeting polarization pathways are favored for their strong operability and broad clinical applicability. However, challenges remain, particularly with respect to side effects and individual variability, as well as the unclear selectivity and long-term effectiveness of these drugs. Gene and cell therapies offer high precision in regulating macrophage polarization, but their clinical translation faces significant barriers due to limitations in technological development and safety concerns. In particular, issues with delivery efficiency and off-target effects remain critical challenges. Nanodrug delivery systems have demonstrated considerable potential in improving targeting and delivery efficiency, which can enhance therapeutic outcomes. However, concerns regarding stability, immune responses, and long-term effects need to be addressed, particularly in the context of large-scale clinical applications. TCM offers the advantage of low side effects and a holistic approach to immune regulation. Nevertheless, its complex mechanisms, variability in efficacy due to individual differences, and challenges with standardization and dosage control hinder its broader clinical implementation. Future research should focus on overcoming these translation barriers by integrating the strengths of different therapeutic strategies. By combining these approaches, multidimensional therapies could be developed to facilitate more efficient cardiac repair and immune regulation, thereby enhancing clinical outcomes.

## 6 Discussion

In the repair process following AMI, macrophage polarization is a highly complex and dynamic process, regulated by the interaction of multiple mechanisms, including metabolism, epigenetics, signaling pathways, and the cardiac microenvironment. These mechanisms not only independently regulate macrophage polarization but also interact and form feedback loops, ultimately shaping macrophage function and determining the immune response and repair outcomes in the heart.

Metabolism and epigenetics are complementary and involve complex feedback mechanisms (69). During macrophage polarization, metabolic changes not only provide the energy needed by cells but also influence epigenetic modifications via metabolic products like acetyl-CoA and lactate (201). For example, glycolysis enhances M1 polarization and influences histone lactylation through lactate accumulation, which further regulates pro-inflammatory gene expression (202). Conversely, fatty acid oxidation supports M2 polarization, regulating histone acetylation via acetyl-CoA to promote anti-inflammatory and repair responses (203). These metabolic products drive macrophage functional transitions through feedback from epigenetic mechanisms.

Additionally, metabolic changes and epigenetic regulation are closely linked with signaling pathway activation (204). Signals from metabolic pathways can directly regulate the activity of key pathways, such as NF-κB and JAK-STAT (205). For instance, metabolic reprogramming activates the NF-κB pathway through HIF-1α accumulation, driving M1 polarization. Simultaneously, the JAK-STAT pathway regulates M2 polarization through cytokine signaling, reducing inflammation and promoting repair. The interaction of metabolic signals, epigenetic mechanisms, and signaling pathways forms a coordinated network that jointly influences macrophage polarization.

The cardiac microenvironment further complicates this dynamic regulation. In the localized environment following heart injury, ECM stiffness and mechanical signals influence macrophage polarization. A stiff matrix environment promotes M1 polarization, while a soft matrix encourages M2 polarization. Mechanical signals, through receptors like integrins, not only affect macrophage morphology and migration but also regulate polarization by altering metabolic states and activating specific epigenetic mechanisms (206). The interaction between mechanical signals, metabolic signals, epigenetic modifications, and signaling pathways allows macrophages to adjust their function in response to environmental changes during tissue repair.

Metabolic reprogramming is considered the most critical mechanism, as it directly provides the energy for macrophages and regulates other mechanisms through metabolic products. It is the foundation of macrophage polarization, influencing gene expression and signaling pathway regulation. The role of signaling pathways, particularly NF-κB and JAK-STAT, is crucial in inflammation and repair. NF-κB is central to M1 activation, but prolonged activation can hinder M2 conversion, delaying repair. Therefore, the regulation of signaling pathways must maintain balance at different stages to ensure coordinated inflammation and repair. The changes in the cardiac microenvironment are secondary but crucial for macrophage polarization through matrix stiffness. These changes are essential during the repair phase, though they may conflict with metabolic and signaling pathway interactions. For example, while a stiff matrix supports M1 polarization, a soft matrix should encourage M2 polarization during repair, leading to potential conflicts between these mechanisms. Finally, epigenetic regulation, though important for long-term macrophage function and stability, has a slower effect and plays a more significant role in later stages of polarization. Thus, its influence is limited during acute immune responses, but it is crucial for maintaining stability during tissue repair.

In conclusion, metabolism, epigenetics, signaling pathways, and the cardiac microenvironment interact to shape the macrophage polarization process. These mechanisms influence each other and ensure macrophages maintain a balance between inflammation and tissue repair at different stages of AMI. Understanding these interactions provides a crucial theoretical foundation for developing multidimensional, integrated treatment strategies and advancing clinical applications for cardiac repair and immune modulation.

## 7 Conclusion and prospects

This review explores the role of macrophages in the repair process after AMI and their polarization regulatory mechanisms. The transition from the pro-inflammatory M1 phenotype to the reparative M2 phenotype is a core process in myocardial recovery, directly influencing tissue damage and subsequent repair. Macrophage polarization is regulated not only by signaling pathways, metabolic states, and microenvironmental changes but also by precise temporal control and other influencing factors. These elements provide crucial insights for gaining a deeper understanding of the cardiac repair process. Understanding these regulatory mechanisms can lay the foundation for developing novel therapeutic strategies to improve myocardial repair and functional recovery after AMI.

Although current research offers preliminary insights into the multidimensional regulatory network of macrophage polarization in AMI, several critical questions remain to be addressed. Specifically, the dynamic changes in macrophage polarization, the regulatory mechanisms in different immune environments, and how these processes impact cardiac repair and functional recovery after AMI need further investigation. Additionally, while research has primarily focused on M1 and M2 polarization, understanding the timing of M1/M2 transitions, the interaction between polarization pathways, and how to achieve precise regulation in clinical settings are key areas for future studies.

From a therapeutic standpoint, regulating macrophage polarization provides a new target for cardiac repair after AMI. Various strategies have been proposed, such as using immunomodulatory drugs, gene editing, TCM interventions, and exosome delivery, all showing potential therapeutic benefits. However, these approaches are still in the experimental phase and need validation through preclinical models and clinical trials to assess their efficacy and safety. Particularly, interventions targeting macrophage polarization may affect immune functions in other parts of the body, so their systemic effects must be carefully evaluated. Since macrophages play vital roles not only in the heart but also in the immune responses of other organs, avoiding potential adverse effects on the overall immune system will be crucial for future research. Moving forward, studies should not only clarify the relationship between macrophage polarization and cardiac repair but also focus on developing personalized treatment strategies that precisely regulate macrophage polarization to enhance cardiac repair and minimize adverse remodeling.

In conclusion, macrophages play a key regulatory role in cardiac repair following AMI, and precisely controlling their polarization offers new possibilities for treating heart disease. With further understanding of macrophage biology, immune regulation, and metabolic mechanisms, along with the application of new technologies, macrophage polarization regulation is expected to become a breakthrough in AMI treatment, providing more precise and personalized therapeutic options for patients.
