# Correction: Macrophage polarization in acute myocardial infarction: multidimensional regulation and emerging therapeutic opportunitiesa

**DOI:** 10.3389/fimmu.2026.2007502

**Published:** 2026-10-07

**Authors:** Zilv Ye, Bo Wang, Zhengdong Wan, Jiawei Guo

**Affiliations:** 1 Department of Vascular and Endovascular Surgery, The First Affiliated Hospital of Yangtze University, Jingzhou, China; 2 Thyroid and Vascular Surgery Department, Jingzhou Hospital Affiliated to Yangtze University, Jingzhou, China; 3 Department of Pharmacology, School of Medicine, Yangtze University, Jingzhou, China

**Keywords:** acute myocardial infarction, immune modulation, inflammatory microenvironment, intervention strategies, macrophage polarization

Author “Jiawei Guo” was erroneously omitted as equal contributing author.

The original version of this article has been updated.

